# A randomized controlled trial: Comparison of 14 and 24 French thoracic drainage after minimally invasive lobectomy – MZ 14-24 study

**DOI:** 10.1016/j.heliyon.2023.e22049

**Published:** 2023-11-07

**Authors:** Davor Stamenovic, Eileen Dittmar, Philipp Schiller, Darko Trenchev, Ioannis Karampinis, Christian Galata, Eric Roessner

**Affiliations:** Department of Thoracic Surgery, Center for Thoracic Diseases, University Hospital Mainz, Mainz University, Germany

**Keywords:** Thoracic surgery, Uniportal VATS, Lung surgery, Lobectomy, Chest tube, Thoracic drainage

## Abstract

**Background:**

The optimal placement of a chest drain after video-assisted minimally invasive lobectomy should facilitate the aspiration of air and drainage of fluid. Typically, a conventional 24Ch polyvinyl chloride chest drain is used for this purpose. However, there is currently no scientific literature available on the impact of drain diameter on postoperative outcomes following anatomical lung resection.

**Methods:**

This is a prospective, randomized, phase-1 trial that will include 40 patients, which will be randomly assigned into two groups. Group 1 will receive a 24 French chest drain according to current standards, while group 2 will receive a 14 French drain. Primary endpoint of the trial is the incidence of postoperative drainage-related complications, such as obstruction, dislocation, pleural effusion, and reintervention. Secondary endpoints are postoperative pain, chest drainage duration, incidence of complications, and hospital length of stay. The study aims to determine the number of subjects needed to achieve a sufficient test power of 0.8 for a non-inferiority study.

**Discussion:**

Thoracic surgery is becoming more and more minimally invasive. One of the remaining unresolved problems is postoperative pain, with the intercostal drain being one of the main contributing factors. Previous data from other studies suggest that the use of small-bore drains can reduce pain and speed up recovery without an increase in drain-related complications. However, no studies have been conducted on patients undergoing anatomic lung resections to date. The initial step in transitioning from larger to smaller drains is to establish the safety of this approach, which is the primary objective of this trial.

Trial registration: The study has been registered in the German Clinical Trials Register.

Registration number: DRKS00029982.

URL: https://drks.de/search/de/trial/DRKS00029982.

## 1 Background

Following video-assisted minimally invasive lobectomy, the placement of a chest drain is a common practice to evacuate air and fluid from the pleural cavity. However, there is a lack of eveidence and randomized studies investigating the optimal drain size for anatomic lung resections and its impact on patient recovery. While some studies support the use of small-bore drains in other lung and thoracic conditions, many surgeons still prefer larger-bore drains due to concerns about potential obstruction [1]. However, bigger drains have been related with increased risk of pain and therefore increased patient discomfort [[2], [3], [4], [5]].

Recent studies [6] have shown no significant difference in complications between small- and large-bore drains, and studies reporting results after pleurodesis [7,8] suggest that small-bore drains are equally effective without an increased risk of obstruction. The use of small-bore drains is already a standard of treatment for conditions like pneumothorax [9], but there is currently no uniform guideline for the appropriate size of thoracic drainage [10]. Similarly, in the treatment of pleural empyema, studies have found no significant difference in disease-associated deaths and required interventions based on drain size, with small-lumen drains being equally effective and associated with less pain [11].

The German Society for Thoracic Surgery Delphi consensus on the management of thoracic drains indicates that a conventional 24 French (F) chest drain is commonly used after lung lobectomy [12]. A recently published nationwide survey confirmed that over 85 % of German thoracic surgeons use 24 F drains or larger [13].

The whole field of thoracic surgery has emensly evolved during the past decade. Complex lung resections are now performed through a 3–4 cm incision. It is contradictory to the principles of minimally invasive surgery to use a drain that is nearly as large as the incision itself, especially considering the very well known relation between chest drains and postoperative pain. Pain still remains one of the unresolved issues following thoracic surgery. Less postoperative pain means better lung function, faster recovery, faster return to normal life. Reducing postoperative pain could positively impact every aspect of our current postoperative pathways on thoracic surgery.

We therefore consider it vital to address this issue in a formal trial. The aim of this phase I trial is to establish the safety of using a small-bore drain in these procedures and gain more insight on the effect of smaller drains on postoperative pain following thoracoscopic anatomic lung resections.

## 2 Methods

### 2.1 Study participants

**Fig. 1:**
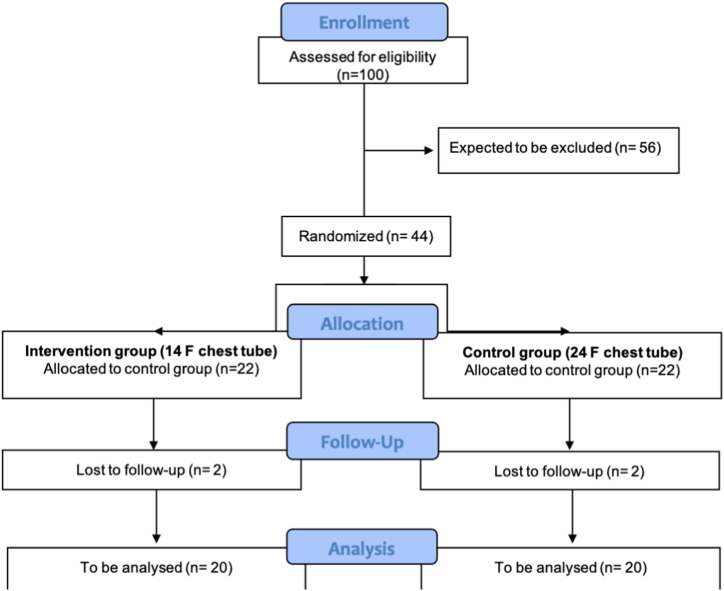
Trial flowchart.

A total of 40 patients undergoing uniportal video-assisted thoracoscopic lobectomy (uVATS) at the Center for Thoracic Diseases (CTD) will be recruited for this study. Patients meeting the inclusion criteria and providing informed consent will be enrolled. The sample size of 40 patients would allow us to analyze 20 patients on every group. This number is considered appropriate for Phase I trials in order to collect the necessary safety information and perform subsequent sample size calculations for a larger efficacy trial. In order to analyze 40 patients and taking into account the increasing number of anatomic segmentectomies performed and our conversion rate, we assume that around 100 patients need to be screened (Fig. 1).

### 2.2 Inclusion criteria

Patients over 18 years of age scheduled to undergo a lobectomy using uVATS for benign or malignant lung lesions, including primary lung cancer or pulmonary metastasis, will be eligible for inclusion in the study.

### 2.3 Exclusion criteria

Patients who do not undergo lobectomy using minimally invasive techniques, and patients undergoing other types of lung resection (wedge resection, segmentectomy, bilobectomy/pneumonectomy) will be excluded. Patients with a history of congenital or acquired coagulopathy, use of direct oral anticoagulants, deviation from standard postoperative pain management regimen, or chronic analgesic therapy will also be excluded.

The study will randomize the 40 patients into two groups of equal size. Group 1 (control group) will consist of 20 patients who will receive a conventional 24 French (24F) chest drain according to current standards. In Group 2, a 14 French (14F) drain will be used (intervention group), which will be placed intraoperatively through the same access required for lung resection. The 14F drain will be secured to the skin with a separate suture, following the same technique as the placement of the 24F drain.

Drain removal is indicated by a board certified thoracic surgeon during the morning round on every postoperative day. In order for the drain to be removed there must not be any air leak during the past 24 h before the morning ward round and the fluid drainage has to be less than 5 ml/kg/24 h [14].

### 2.4 Trial timeline

The recruitment phase has been estimated at 12 months. The overall trial duration will be two weeks after the last patient has been discharged.

## 3 Assessment of outcomes

### 3.1 Primary outcome measures

Drain related complications: includes irrigation of the drain, repositioning of the drain, placement of an additional drain, re-VATS (video-assisted thoracoscopic surgery), placement of a new drain after previous drainage.

### 3.2 Secondary outcome measures

• Postoperative pain (Postoperative pain levels will beassessed twice daily (BID) during the first three days postoperatively).
• Drainage time in days (rounded to the nearest whole number).
• Length of hospital stay in days (rounded to the nearest whole number).
• Postoperative complications

These parameters will be collected as part of the clinical routine. Additional patient data will include the type of surgery (specific lobe), pre-disease profile, laboratory parameters, and pre- and postoperative radiological and clinical findings. The preoperative preparation, diagnostic procedures, and pre-admission process will follow the standard clinical routine without any deviations.

## 4 Implementation

The physicians at the Center for Thoracic Diseases (CTD) will strictly follow the established clinical routine for surgery preparation, diagnostics, and pre-admission procedures. Informed consent will be obtained from all participating patients by the physicians. Patients who provide consent for surgery will be informed about the opportunity to participate in the study, provided they meet the inclusion criteria and have no exclusion criteria. During the consenting process the benefits and risks will be thouroughly explained, and enrollment will proceed after written consent has been obtained.

All referrals for surgery are discussed in the local multidisciplinary team meeting. The choice of the surgical approach is always determined by a board certified thoracic surgeon. All surgical procedures will be performed by or supervised by a board certified thoracic surgeon with experience in uniportal thoracoscopic lung resections (>100 thoracoscopic anatomic lung resections). Three consultant surgeons fulfill these criteria in our department. The treatment method implemented will be based on randomization, with one group receiving 14F drainage and the other group receiving 24F drainage. The consultant surgeon performing or supervising the procedure will be responsible for deciding if the patient is eligible for randomization. It is our institutional practice to ventilate the lung at the end of the procedure and perform an underwater air leak test in order rule out major air leaks and check the bronchial closure.

If the thoracic consultant considers the patient eligible for randomization, the randomization process will take place before insertion of the drain at the end of the procedure. The drains will be inserted through the surgical incision into the chest cavity, and their positioning will be visually confirmed before securing them to the skin using a suture made with Supramid 0 thread from B.Braun. Standardized sutures will be used to close the muscle, subcutaneous tissue, and skin. Both groups' drains will be connected to a digital thoracic drainage system (Thopaz from Medela Healthcare, Switzerland) with a suction of −8 cm H2O (physio mode). All electronic suction devices will be calibrated before use as recommended by the manufacturer.

Daily clinical data will be collected, including the fever curve to monitor body temperature, pain intensity using the Numerical Analog Scale (NAS) recorded twice daily, analgesic medication administered, quantity and quality of secretion per 24 h (recorded by nursing staff at 6 a.m.), and assessment of parenchymal fistula (recorded during the morning visit at 7:45 a.m. based on the graph displayed in the last 24 h). Incision wound checks will be performed on the day of surgery, at discharge, and during follow-up visits at the outpatient clinic, usually scheduled within 14 days after discharge.

Postoperative procedures, including drainage and pain management, will follow our standard operating procedure for postoperative care after thoracic surgery. Patients following anatomic lung resections are usually monitored for 24 h on a high dependency or intensive care unit. If the immediate postoperative period is uneventful the patients are usually transferred on the ward on the first postoperative day. The patients are usually discharged one day after drain removal. A final follow-up visit is part of our postoperative care standard operating procedure and usually takes place 2 weeks after surgery in the outplatient clinic.

As part of routine clinical practice, patients typically receive three peripheral nonsteroidal analgesics and a perivertebral pain catheter with 0.375 % Ropivacain at a rate of 10 ml/h. A chest X-ray (PA view) will be performed on the first postoperative day, immediately prior to drainage removal, and during follow-up visits at the outpatient clinic. If the X-ray shows any pathological findings, further tests may be requested as indicated.

Before discharge, patients are informed to visit the Department of Thoracic Surgery if they experience any complaints.

A risk-benefit analysis has determined that the placement of a postoperative chest drain is a routine procedure without additional burden to the patient. While treatment with a 24F chest drain is a standard procedure with no increased risk, treatment with a 14F chest drain may result in more postoperative interventions due to potential complications such as pneumothorax or drain occlusion. However, the medical staff at CTD is highly senzibilized to these possibilities and prepared to promptly react if such events occur. After the surgery, all patients are monitored on the intermediate care unit overnight under close supervision by the medical staff.

Patients in the experimental group may experience less pain, reduced morbidity, shorter hospital stays, and increased satisfaction.

### 4.1 Drop-out

There are multiple factors that can result in the discontinuation of an individual's participation in the study, such as the participant's voluntary withdrawal or failure to meet the inclusion and/or meeting the exclusion criteria. However, it is important to note that these circumstances do not fall within the category of drop-outs, since no randomization takes place prior to these occurrences.

### 4.2 Safety

If the rate of complications or the need for renewed drainage of the pleural cavity in the experimental group exceeds 20 % among the first ten subjects, the study will be discontinued.

### 4.3 Biometry

The collected measurement data will be described using descriptive statistics and compared with routine clinical data from both groups. The goal is to determine the number of cases in each group for a “non-inferiority” study to assess the risk of complications in the experimental group using a 14F chest tube.

This study is a prospective phase I trial that involves randomization of participants using an “envelope” randomization method. A third-party non-physician writes either 14 or 24 on 40 sheets, which are then placed in sealed envelopes. During the chest drain placement procedure, a random envelope is selected to determine the size of the chest drain.

The collected data will be analyzed using descriptive statistics initially. Subsequently, exploratory analyses will be conducted to compare groups and identify risk factors. Statistical methods such as t-tests, Mann-Whitney U-tests, analysis of variance, or Kruskal-Wallis tests will be used based on the sample size, scale level, and variance of the parameters. If necessary, multivariable test methods like logistic regression or multiple linear regression will be employed to assess the influence of multiple variables on an outcome variable.

### 4.4 Data management and protection

The data collected will be stored digitally on data storage devices at the Thoracic Surgery department of the University Medicine Mainz. Data security will be ensured by following the data protection guidelines of the University Medical Center Mainz and complying with legal requirements, including the General Data Protection Regulation (GDPR).

The study will be conducted in accordance with the current version of the Declaration of Helsinki. The study protocol has been approved by the Ethics Committee of the district medical council of Rhineland-Palatinate (approval Nr. 2021–15994). All patient information, including names and other confidential data, will be treated with medical confidentiality and will be subject to the provisions of the State Data Protection Act of Rhineland-Palatinate and the Federal Data Protection Act (LDSG BW and BDSG). Patient data will only be shared in pseudonymized form, and original documents will not be accessible to third parties.

The data will be stored for a period of 25 years, in compliance with the provisions of the Good Clinical Practice (GCP) regulation of August 9, 2004. Participation in the study is voluntary, and patients have the right to withdraw their consent at any time without providing a reason and without any negative impact on their medical care. Before the study commences, participants will be provided with written and verbal information about the nature and scope of the planned investigation, including potential benefits and risks to their health. Their consent will be documented through the signing of an informed consent form. In the event of withdrawal from the study, any data already collected will be destroyed, or the patient will be asked for permission to analyze the existing materials.

## 5 Discussion

After thoracic surgery, chest drains are commonly used to facilitate the drainage of fluids and air and provide information about the thorax. However, the conventional chest drain placement can cause discomfort and affect breathing and secretion mobilization, potentially leading to complications such as dystelectasis, atelectasis, or pneumonia.

The smallest drainage diameter is not represented by the lumen of the actual tube, but by the connector diameter. This is the same from 12 Ch to 20 Ch. Therefore, chest drains are divided into two categories based on their sizes: large-lumen (≥20Ch) and small-lumen (≤20Ch) drains.

In a previous experimental study [15], the drainage capacity of a 19F chest drain was compared to a 28F chest drain under both in vivo and in vitro conditions. The study found that the larger-bore drain had a nine-fold increased capacity in vitro, meaning it was able to drain fluid at a much higher rate compared to the smaller-bore drain. However, interestingly, when tested in vivo conditions, both drains demonstrated the same drainage capacity.

Another study [16] focused on the relationship between drain size, fluid viscosity, and flow rate. The study found that there were significant differences in flow rates among catheters with sizes of 8F or smaller, indicating that smaller-bore drains had reduced flow rates compared to larger-bore drains. While large-bore drains offer increased drainage capacity, they are also associated with higher levels of pain. On the other hand, small-bore drains have a higher risk of obstruction or kinking, requiring removal and possible repositioning, which can cause additional discomfort for the patient. Statistically, the risk of obstruction is reported to be 8.1 % with small-bore drains compared with 5.2 % with large-lumen drains [17].

There is currently no standard guideline for managing intraoperative and postoperative drainage after lobectomies due to limited evidence and varying opinions among surgeons [18]. However, the implementation of Enhanced Recovery After Surgery (ERAS) protocols has sparked research focusing on aspects such as the optimal timing of chest drain removal, the number of drains used, the use of suction, and the selection of appropriate drainage systems. Unfortunately, there is a lack of studies specifically dedicated to investigating the impact of drainage lumen size, with limited research available on this topic [[19], [20], [21]].

There is evidence supporting the use of small-bore chest drains in the therapy of empyema, showing equivalent effectiveness compared to large-bore drains [22]. In fact, small-bore, CT- or US- guided drains are currently widely used for the treatment of pleural empyema worldwide. Small-bore drains have also been associated with reduced pain symptoms and faster recovery in patients undergoing thoracic surgery [2,23].

Early removal of chest drains has been shown to contribute to shorter hospital stays and better postoperative recovery [24]. Studies have even compared the use of urinary bladder catheters to chest drains and found that catheters resulted in shorter time to drain removal, reduced hospital stay, and improved wound healing [25].

Based on the these studies, small-bore drains have demonstrated safety in pre-clinical studies and in patients undergoing surgery for other thoracic conditions. Our hypothesis is that if a small-bore drain is safe and effective in treating pleural empyema, it should be more than sufficient for draining serous fluid and air leaks following a uniportal VATS lobectomy.

## 6 Limitations

The purpose of this study is to examine safety in this specific patient population. In addition, the dataset generated during this trial should enable the sample size calculation for a subsequent feasibility trial. The limitations of this trial mainly result from the monocentric character of our study. This could limit the generalisability of our findings. Furthermore, we intentionally decided to include a patient cohort consisting of both patients with primary lung cancer and metastatic disease in order to face the current practice in thoracic surgery. Due to the nature of this trial we were not able to stratify for lung function. This could theoretically lead to an unbalanced distribution of patients with regards to lung function between the two groups. However, we are planning to address this issues upon completion of the trial and take them into consideration during the planning phase of the following trial.

## 7 Potential benefits of the trial

As reported above, this is a phase I trial. It is only designed to prove safety and allow the sample size calculation for a following trial. During this trial we are planning to collect primary data on postoperative pain for patients undergoing anatomic lung resections. If the results of this trial confirm the safety of the smaller drains and suggest lower pain scores in the intervention group we would perform a further, large scale trial with this endpoint. Resolving the postoperative pain issue is one of the biggest challenges in modern thoracic surgery. This phase I trial could be the first step towards solving it.

## Ethics approval and consent to participate

All methods were carried out in accordance with relevant guidelines and regulations and approved by the district medical council of Rhineland-Palatinate (Approval Nr. 2021–15994). Informed consent will be obtained from all subjects and/or their legal guardian(s).

## Consent for publication

Not Applicable.

## Availability of data and materials

This paper here presents the trial protocol and there are therefore no datasets to make available for further use. The raw data that will be collected during this trial will be subject of a second publication in the future.

## Funding

None.

## CRediT authorship contribution statement

**Davor Stamenovic:** and, made significant contributions to the writing of the manuscript. **Eileen Dittmar:** and, collected the data and obtained the necessary approvals, All authors have thoroughly reviewed and approved the final version of the manuscript. **Philipp Schiller:** and. **Darko Trenchev:** and. **Ioannis Karampinis:** conceptualized the study, were responsible for analyzing and interpreting the patient data. **Christian Galata:** and, and. **Eric Roessner:** and.

## Declaration of competing interest

The authors declare that they have no known competing financial interests or personal relationships that could have appeared to influence the work reported in this paper.

## List of abbreviations

F: French
CTD: Center for Thoracic Disease
VATS: video-assisted thoracoscopic surgery
BMI: body-mass index
NAS: numerical analog scale
ERAS: enhancement recovery after surgery
FEV1: forced expirium volume in the first second
